# Improving global health security through implementation of the National Action Plan for Health Security in Sierra Leone, 2018–2021: lessons from the field

**DOI:** 10.1186/s12889-023-17103-7

**Published:** 2023-11-07

**Authors:** Charles Njuguna, Mohamed Vandi, Tushar Singh, Ian Njeru, Jane Githuku, Wilson Gachari, Robert Musoke, Victor Caulker, Joseph Bunting-Graden, Michael Mahar, Sydney Morgan Brown, Mohamed Alpha Bah, Mo-Bashir Idriss, Ambrose Talisuna, Dick Chamla, Zabulon Yoti, Rajesh Sreedharan, Ludy Suryantoro, Abdou Salam Gueye, Stella Chungong

**Affiliations:** 1 WHO Country Office for Sierra Leone, 21 A & B Riverside Drive, off King Harman Road, Brookfield, Freetown, Sierra Leone; 2 Ministry of Health and Sanitation, Freetown, Sierra Leone; 3 US CDC, Atlanta, Georgia; 4 Ministry of Agriculture, Freetown, Sierra Leone; 5 Ministry of Environment and Climate Change, Freetown, Sierra Leone; 6 WHO Regional Office for Africa, Brazzaville, Congo; 7 WHO Geneva, Geneva, Switzerland

**Keywords:** International Health Regulations, Health security, National action plan

## Abstract

**Background:**

All countries are required to implement International Health Regulations (IHR) through development and implementation of multi-year National Action Plans for Health Security (NAPHS). IHR implementation requires annual operational planning which involves several tools such as NAPHS, State Party Annual Report (SPAR), Joint External Evaluation (JEE) and WHO IHR Benchmarks tool. Sierra Leone has successfully improved IHR capacities across the years through successful annual operational planning using the above tools. We conducted a study to document and share the country’s unique approach to implementation of NAPHS.

**Methods:**

This was an observational study where the process of implementing and monitoring NAPHS in Sierra Leone was observed at the national level from 2018 to 2021. Data was obtained through review and analysis of NAPHS annual operational plans, quarterly review reports and annual IHR assessment reports. Available data was supplemented by information from key informants. Qualitative data was captured as notes and analysed for various themes while quantitative data was analyzed mainly for means and proportions.

**Results:**

The overall national IHR Joint External Evaluation self-assessment score for human health improved from 44% in 2018 to 51% in 2019 and 57% in 2020. The score for the animal sector improved from 32% in 2018 to 43% in 2019 and 52% in 2020. A new JEE tool with new indicators was used in 2021 and the score for both human and animal sectors declined slightly to 51%. Key enablers of success included strong political commitment, whole-of-government approach, annual assessments using JEE tool, annual operational planning using WHO IHR Benchmarks tool and real time online monitoring of progress. Key challenges included disruption created by COVID-19 response, poor health infrastructure, low funding and inadequate health workforce.

**Conclusion:**

IHR annual operational planning and implementation using evidence-based data and tools can facilitate strengthening of IHR capacity and should be encouraged.

## Background

According to International Health regulations (IHR 2005), all World Health Organization (WHO) member states are obliged to build public health capacities to prevent, detect and respond to potential health threats as well as adhere to reporting requirements on public health threats [1]. Where public health capacities are under-developed, epidemics such as the Ebola virus disease (EVD) outbreak in West Africa (2014–2016) and the ongoing COVID-19 pandemic result in loss of many lives, weak health systems, and heavy economic losses [2–4].

The Severe Acute Respiratory Syndrome (SARS) pandemic of 2003 and other past outbreaks provided the necessary impetus for accelerated revision and adoption of the International Health Regulations (2005) [5]. This widened the scope of reportable health events and provided an opportunity to increase core public health capacities in all state parties. Unfortunately, compliance with IHR regulations was slow with only 42/196 (21%) member states reporting compliance in 2012 which was the first deadline for countries to have built the required IHR capacities. Even after the compliance period was extended to 2014, only 64/196 (33%) states were found to be compliant [6].

Sierra Leone was among the countries that had not complied to IHR (2005) by the time the EVD outbreak started in 2014 [7], and this was among the factors that contributed to the rapid spread of the outbreak. Besides Ebola, other recent public health threats in Sierra Leone include Lassa fever, cholera, rabies and mudslide related health issues [8–11]. A decade of civil war (1991–2002) also resulted in under investment in the health sector. Additionally, in the post conflict period, provision of basic health services was prioritized over public health [12, 13] and public health core capacities development lagged behind.

### Moving towards improved global health security post EVD outbreak

The EVD outbreak awakened the world to the need for better global preparedness and response to public health emergencies. Initiatives such as the global health security agenda (GHSA) seeking to accelerate the implementation of IHR were launched [14]. The World Health Organization also developed several tools to support countries in building their capacities for IHR. These tools include the Joint External Evaluation (JEE), National Action Plan for Health Security (NAPHS) and the IHR Benchmarks tool. While many countries in the world have struggled to successfully use these tools, Sierra Leone has adopted a unique implementation approach that uses many of these tools. In this paper we share the unique experience of implementing the tools in Sierra Leone and how this approach led to improvement in IHR capacities from 2016 to 2021.

### The Joint External Evaluation

The World Health Assembly through resolution WHA68.5 in May 2015, adopted a shift in monitoring of IHR implementation from exclusive self-evaluation to approaches that combine self-evaluation, peer review and voluntary JEE involving a combination of domestic and independent experts [15]. The JEE examines compliance with IHR and emergency preparedness using indicators across 19 technical areas. Each of the indicators is assigned a score between one and five; the score of a one is used when a country has no documented capacity in the given indicator, while a five is used when the country has demonstrated strong sustained capacity in the given indicator.

Having suffered the largest number of casualties in the EVD outbreak [16], Sierra Leone was eager to recover from the effects of the outbreak, reduce susceptibility to epidemic diseases and improve future emergency response [13, 17]. This meant addressing the gaps that were found to be responsible for the prolonged outbreak which were mainly weak health systems, inadequate basic infrastructure, and under-developed public health capacities. Sierra Leone was therefore the sixth country to volunteer for the JEE in 2016, through which a number of recommendations for improving health security were made [18].

### Developing a National Action Plan for Health Security for Sierra Leone, post-JEE

The preparedness cycle in relation to health security involves planning, implementation, assessment, realignment, and evaluation. In line with this model, state parties that have successfully undergone JEE, are expected to develop national action plans for health security that address the recommendations of the JEE using all hazard and One Health approaches. Following the JEE, WHO, US Centers for Disease Control and Prevention (US CDC) and other partners supported the Ministry of Health and Sanitation (MoHS) to develop the NAPHS [19].

The costed action plan outlining priority health security preparedness activities for 2018–2022 was completed in 2018. The estimated cost of implementation was 291 million US dollars, with 50 million dollars required for implementation of high priority activities in the first two years. Next, the country used a resource mapping tool (REMAP) developed by WHO headquarters to identify funding sources and gaps for NAPHS implementation [20].

## Methods

### Study design and setting

The Sierra Leone NAPHS was developed in 2018 and was implemented over 5 years (2018–2022). This study examines the impact of further utilizing annual operational plans to implement the NAPHS and to improve internal JEE and State Party Annual Report (SPAR) self-assessment scores.

### Data collection and analysis

Data for this paper was obtained through review and analysis of key documents that were developed or compiled during the implementation of NAPHS from 2018 to 2021 (NAPHS annual operational plans, quarterly review reports, annual JEE self-assessment reports and annual SPAR reports. Annual JEE self-assessments used the WHO JEE tool [21] while the SPAR assessments used the WHO SPAR tool [22]. The annual NAPHS operational plans used a tool that was devised by the country. Since the JEE/SPAR assessments and the annual operation plans provided the bulk of the data for this paper, the two processes have been described in detail later in this section.

The data obtained from the assessments and operational plans was supplemented by information from key informants from the key stakeholders such as the NAPHS technical area focal persons from the various government ministries, departments and agencies (MDAs). Other key informants were from key development partners such as WHO and US CDC.

Quantitative data was analyzed mostly for means (continuous variables) and proportions (categorical data). Qualitative data was captured as notes and analyzed for relevant themes around the topics related to the NAPHS technical areas.

### Iterative annual assessments of health security capacity using SPAR and JEE tools

Annual completion of IHR SPAR report is mandatory for all WHO member states and the report is submitted to WHO. In Sierra Leone, this exercise is conducted annually at the end of the year, using the SPAR tool. Government officials from human health sector, animal health sector, environment sector and Office of National Security (ONS) with support of WHO, US-CDC and other partners, holds 2–3 days’ consultative workshops to deliberate on this annual SPAR report.

During the SPAR workshop, the participants also conduct a voluntary internal self-assessment using the JEE tool although the actual JEE is conducted every 4–5 years. The JEE tool is more comprehensive and has more indicators than the SPAR tool. Following the assessment, a comprehensive JEE self-assessment scorecard report is then compiled annually.

The workshop includes participants from each sector, representing each of the 19 NAPHS technical areas. Participants are divided into groups representing 3–5 technical areas each. Each group jointly discusses each JEE and SPAR indicator (where different), progress that has been made over the course of the year, and what evidence they have to support each given score. During the last day of the workshop, a representative of each technical area presents the discussion outcome and the recommended score to a plenary session of all participants for consensus. These scores are used both to measure progress (see results section) and to develop the annual operational plan.

### Development of annual operational plans

The country’s NAPHS 5 years’ plan (2018–2022) had 48 objectives, 167 strategic activities and more than 900 sub activities. Following the annual assessments (SPAR and JEE), Sierra Leone holds a 2–3 days’ planning workshop at the beginning of each year to develop the annual operational plan (AOP) for NAPHS. The participants from the 19 technical areas are drawn from government MDAs with facilitation support from partner organizations. During the planning workshop, the country uses a unique approach to select a few priority activities from each of the 19 technical areas.

**Fig. 1:**
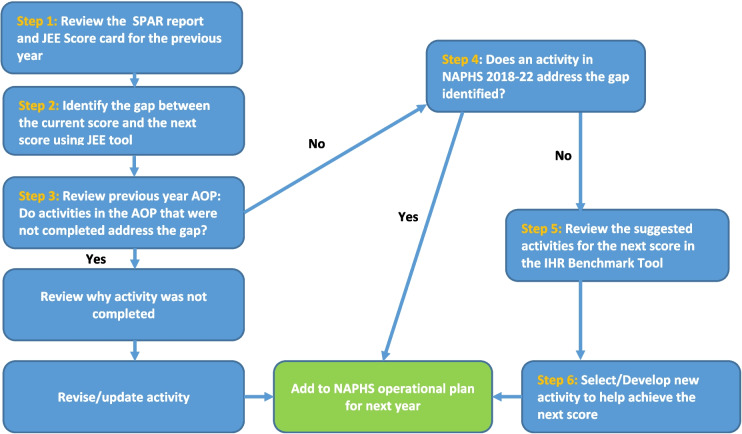
Process for selecting activities for the NAPHS annual operational plan, Sierra Leone

To select priority activities for the AOP, several documents are used: the annual SPAR report, JEE self-assessment score card, JEE assessment tool, AOP for the previous year, NAPHS 2018–2022 and WHO IHR Benchmarks tool. The first step in selecting NAPHS activities for inclusion in the AOP is to review the SPAR report and JEE score card for the previous year. Step 2 is identifying the gap between the current score and the next score using JEE assessment tool and JEE scorecard. Step 3 is to review previous year AOP to check if there are activities that were not completed but address the gap. If yes these activities are reviewed/revised and added to the operational plan. Step 4 is to review the NAPHS 2018–22 plan and check if there are activities that address the gap identified. If yes these activities are added to the operational plan. Step 5 and 6 is to review and select suggested activities from the Benchmark Tool if they help to address the gap (Fig. 1).

Since the activities that require to be implemented for the score to improve during the next round of SPAR & JEE assessments may be many and funding may not be available to cover the implementation of all of them, the country team only prioritizes a few activities per technical area (3–4 activities on average). In 2019, a total of 52 activities were selected for the AOP from the 19 technical areas while 64, 70 and 73 activities were selected in 2020, 2021 and 2022, respectively. The benchmark tool is used to guide which activities should be implemented first in addressing the gap in a certain indicator. If there are many activities for an indicator to improve, some are pushed to the following year.

Tracking progress is a critical component of the NAPHS implementation process in Sierra Leone. Once an AOP is developed, it is then uploaded online for real time monitoring of implementation. To be able to track progress from the 19 technical areas, the country has identified leads for all the technical areas. These technical leads regularly update the status of planned activities. Quarterly review meetings are also held comprising of all the technical leads as well as other NAPHS stakeholders.

## Results

The JEE tool assesses more IHR capacities and indicators as opposed to the SPAR tool. Therefore, the findings presented here are those from the JEE annual self-assessments.

### Trend in IHR capacities: 2018–2021

**Fig. 2:**
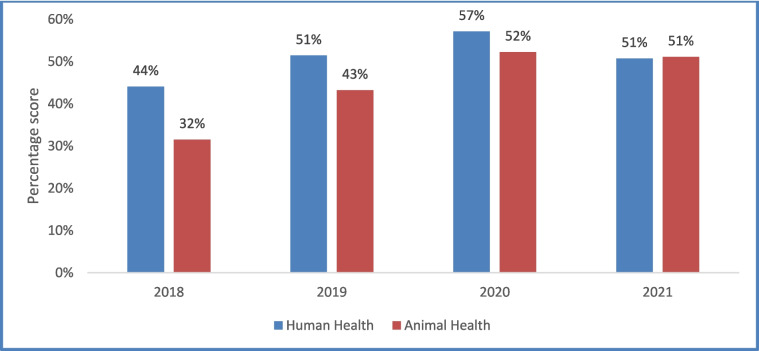
Trend of Joint External Evaluation self-assessment overall percentage score by sector, Sierra Leone, 2018–2021

Overall, moderate improvements were observed in IHR capacities from 2018 to 2021. Using the JEE tool (2018 edition), the overall JEE score for human health improved from 44% in 2018 to 51% in 2019 and 57% in 2020. A similar improvement trend was observed in the animal sector where the score improved from 32% in 2018 to 43% in 2019 and 52% in 2020. In 2021, a new and more stringent JEE tool (2022 edition) with revised and new indicators was used and therefore the overall scores dropped slightly to 51% in both human and animal sectors (Fig. 2).

**Fig. 3:**
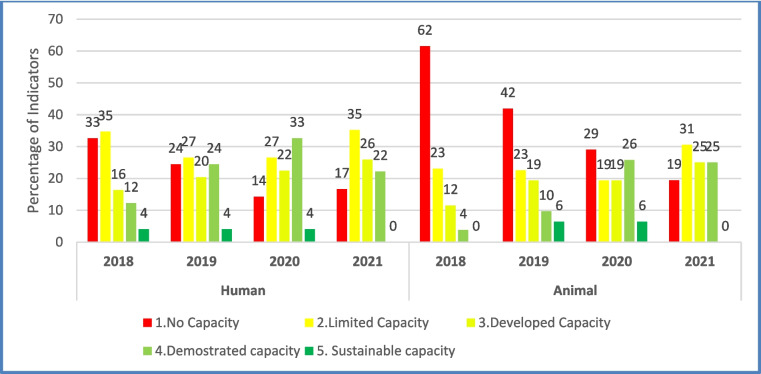
Trend of Joint External Evaluation self-assessment indicator performance by sector, Sierra Leone, 2018–2021

The IHR capacity for each JEE indicator has five levels: No capacity (score 1), Limited capacity (score 2), Developed capacity (score 3), Demonstrated capacity (Score 4) and Sustainable capacity (score 5). The IHR capacity for the country improved steadily from 2018 to 2020 with many of the indicators moving from a lower capacity level (Score 1 and 2) to a higher level (score 3–5) as shown in Fig. 3. For example, the number of indicators that had no capacity (score 1) in the human health sector was 33% in 2018, 24% in 2019, 14% in 2020 and 17% in 2021. Similarly, in the animal health sector, the number of indicators that had no capacity (score 1) was 62% in 2018, 42% in 2019, 29% in 2020 and 19% in 2021. The number of indicators that had demonstrated capacity (score 4) in the human health sector was 12% in 2018, 24% in 2019, 33% in 2020 and 22% in 2021. Similarly, in the animal health sector, the number of indicators that had demonstrated capacity (score 4) was 4% in 2018, 10% in 2019, 26% in 2020 and 25% in 2021. The slight drop in performance trend in 2021 was attributed to the use of the new JEE tool (2022 edition) that made scores in several indicators not directly comparable to previous years.

In the 2021 assessment, no indicators in human health had sustainable capacity (score 5), 22% had demonstrated capacity (score 4), 26% had developed capacity (score 3), 35% had limited capacity (score 2) and 17% had no capacity (score 1). In animal health, no indicators had sustained capacity (score 5), 25% had demonstrated capacity (score 4), 25% had developed capacity (score 3), 31% had limited capacity (score 2) and 19% had no capacity (score 1) (Fig. 3).

### IHR capacity by technical areas

**Fig. 4:**
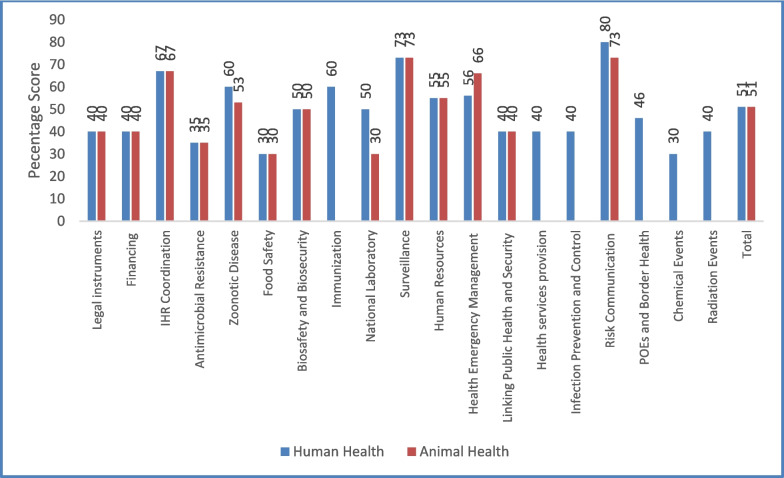
JEE Self-assessment percentage scores by technical area and sector, Sierra Leone, 2021

IHR Capacity improved in most technical areas between 2018 and 2021 in both human and animal health sectors. Data from 2021 IHR assessments showed that human health sector was slightly better than animal health in some indicators although the overall score was the same at 51%. Some of the best performing technical areas included risk communication and community engagement, surveillance and IHR coordination. Poorly performing technical areas included food safety, chemical events, antimicrobial resistance, and radiation events (Fig. 4).

The specific areas that are lagging behind for poorly performing technical areas have been highlighted in Table 1.

**Table 1:** Summary of issues contributing to low IHR capacity in Sierra Leone as at December 2021

**Technical Area**^a^	**Issues Contributing to Low capacity (No capacity and Limited Capacity)**
Legal instruments	• Delay in enacting revised and enabling legislation for IHR implementation in both human and animal health sectors • No systematic assessment of gender gaps in any of the IHR capacities had been conducted
Financing	• Inadequate sustainable funds for IHR implementation and public health emergency response
Antimicrobial Resistance	• Antimicrobial resistance (AMR) surveillance system not in place • No strategy for Prevention of Multi-Drug Resistant Organism (MDRO) • Lack of AMR policy on antimicrobial use in humans and animals
Food Safety	• Weak surveillance and response system for food borne events
Biosafety and Biosecurity	• Weak biosafety and biosecurity system
National Laboratory Systems	• Limited capacity to test priority diseases for animal health • Inadequate sample transport and referral system in human and animal health sector • Lack of a national body for licensing, inspection, accreditation of laboratories
Human Resources	• Inadequate human resources for IHR implementation at the sub-national level in both human and animal sectors
Health emergency management	• A capacity/readiness assessment for potential public health emergencies had not been conducted in the past 2 years
Health service provision	• National clinical case management guidelines for priority health events not in place for some conditions/events
Infection Prevention and Control	• There was no national Health Care Associated Infection(HCAI) surveillance programme or national strategic plan for HCAIs surveillance • Inadequate Water, Sanitation and Hygiene (WASH) infrastructure in health facilities
Points of Entry	• Contingency plans for all designated points of entry (ground crossings and sea port) had been developed but not fully implemented
Chemical Events	• Weak surveillance system for detection and response to chemical events • Limited access to laboratory diagnostic capacity to confirm chemical events
Radiation Emergencies	• Policies and strategies for the detection, assessment and response to radiation emergencies had been developed but not fully implemented

^a^Technical Areas are based on the revised Joint External Evaluation tool (2022 Edition)

## Discussion

Capacity for IHR improved steadily in Sierra Leone from 2018 to 2021. Several factors facilitated this improvement which was largely due to successful planning and implementation of NAPHS. In this section, we discuss how coordination and financing was managed as well as the enablers and challenges that contributed to the results obtained.

### Coordination of NAPHS

Successful implementation of NAPHS requires a well-articulated multi-sectoral and multidisciplinary coordination mechanism. Unfortunately, this is a major gap in most African countries as identified in their JEE [23]. Coordination was also a major gap identified in the 2016 JEE in Sierra Leone and there was no structured IHR focal point that could fulfil the requirements of IHR (2005) [18]. A structure was therefore developed to coordinate implementation of IHR and NAPHS at the national level.

**Fig. 5:**
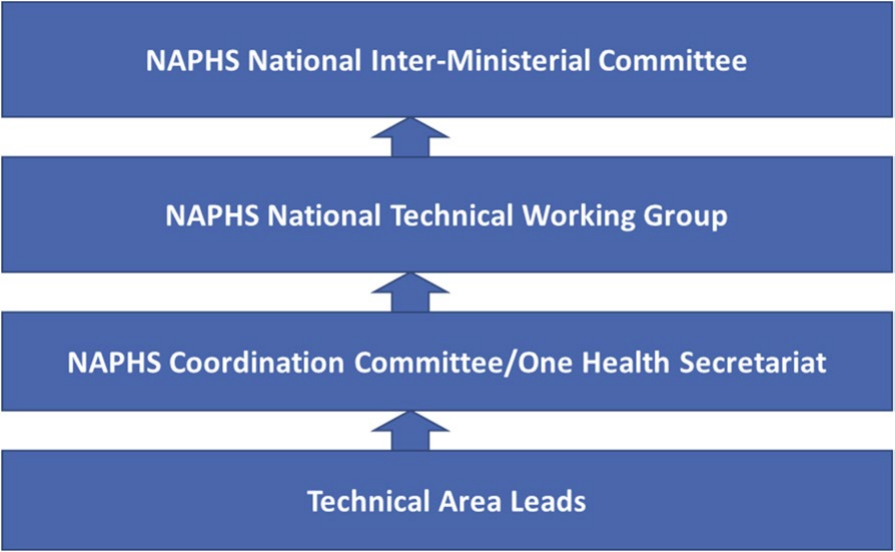
NAPHS coordination structure in Sierra Leone

The overall coordination of NAPHS implementation in the country is done by the NAPHS coordination committee which comprises of the IHR National Focal Point and the One Health Secretariat. This coordination committee is responsible for liaising with the technical area leads from various ministries, departments and agencies. The technical area leads are responsible for coordinating activities in their technical areas both at national and subnational level. The coordination committee reports to the NAPHS multi-sectoral technical working group (TWG) that has technical area focal persons from all the 19 technical areas as well as other stakeholders like development partners. The TWG holds quarterly review meetings to review progress. The TWG reports to the National Inter-Ministerial Committee which meets twice a year and comprises of the Government Ministers relevant to IHR (Fig. 5). This model of coordination seems to work well in Sierra Leone and could be adapted in other countries.

### Financing of NAPHS

Financing of NAPHS is a major challenge for most countries mostly because it cuts across several sectors with different capacities for resource mobilization [23]. The NAPHS operational plan that is developed each year is the main resource mobilization tool for Sierra Leone. The multi-sectoral coordination meetings (quarterly NAPHS review meetings) and the annual SPAR and JEE self-assessments were mainly funded by the World Health Organization. Each sector technical area focal person was responsible for mobilizing resources from domestic and external sources to fund their activities in the operational plan.

Inadequate funding was a major challenge since there was no central fund for NAPHS. This made some technical areas that do not have many or strong funding sources to lag behind. These includes, inter alia, Radiation events, Chemical events and Food safety. The low capacity in these technical areas poses a great morbidity and mortality risk for the country should a serious emergency involving food, chemical and radiation events arise. A mechanism should therefore be put in place in the country to ensure adequate and sustainable funding for all technical areas. This would therefore benefit from further research on the best modalities for funding NAPHS.

### Enablers of success

Several factors were responsible for the successive NAPHS implementation in Sierra Leone and we highly recommend them to other countries. This included a strong political commitment as demonstrated by the launch of the NAPHS by the President of Sierra Leone in 2019 [24]. There was also keen follow up of NAPHS implementation by the various Government Ministers responsible for various sectors. A strong multi-sectoral coordination mechanism using the One Health approach ensured there was involvement and engagement of various stakeholders in government, non-governmental organizations, non-state actors, civil society and development partners.

Sierra Leone was the first country in the African region to use the JEE tool for self-assessment on a regular (annual) basis in addition to the mandatory SPAR tool. Openness to external evaluation and annual internal assessments allowed better understanding of the true status of health security in Sierra Leone and hence fostered evidence-based planning. WHO facilitated NAPHS development and implementation process by availing new tools such as the JEE 2^nd^ edition (2018), JEE 3^rd^ Edition (2022), Benchmark tool and REMAP tool for resource mobilization. The country has been using these tools for the annual operational planning process and this has made annual IHR assessments and operational planning easier to carry out.

Another success factor was the nomination of NAPHS technical area leads for all the 19 technical areas with clear written terms of reference on their roles and responsibilities. The technical area leads now act as implementation catalysts by ensuring that follow up on action points is made in all technical areas. These technical area leads also help in updating the activities in the online NAPHS implementation monitoring platform which ensures real time information and dashboards are available to stakeholders.

Activities with champions from development partners and those with multiple funding sources also tended to be completed earlier than others. We also noted that where complex tasks were involved such as developing a multi-hazard preparedness and response plan, use of consultants to provide extra technical assistance helped to achieve that quite easily and faster.

### Challenges

Despite the gains made in IHR capacity, several challenges limited the scope of success in some of the technical areas. Apart from the financial challenges described above, NAPHS implementation was negatively affected by the COVID-19 pandemic [25]. Many of the planned activities in 2020 were therefore not implemented due to re-direction of human resources and funding to COVID-19. A NAPHS review that was done in the country in June 2021 showed that 51% of the planned activities in the country had been completed against an expectation of 70% after three and half years of implementation. The situation later improved and implementation in late 2021 was largely back on track although at a slower pace. These lessons learnt from COVID-19 calls for better planning and more resilient systems that can withstand future pandemics without much disruption to routine programs.

NAPHS implementation was also affected by inadequate human resource. During the EVD outbreak in West Africa 2014–2016, a total of 211 health care workers died in Sierra Leone, thus reducing availability of health care workers in a country that already had shortages of health care workers [13, 26]. In 2020, it was estimated that Sierra Leone had the lowest density of medical doctors in West Africa at only 3 physicians per 100,000 individuals [27]. This shortage of human resource affected all IHR sectors and is a major hindrance to implementation of planned activities at all levels including national, district and health facility level. At national level, attendance of IHR meetings was often poorly attended by some sectors due to competing tasks. To address this challenge, the government need to continue putting measures in place to increase human resource capacity in all sectors.

IHR requires progressive improvement of capacities both at national and subnational level. This requires supportive infrastructure which has been a challenge for Sierra Leone. The country was among the least developed countries in the world in 2018 and ranked 184 out of 189 in terms of human development [28]. Infrastructural development stalled during the period of civil unrest and was worsened by the EVD outbreak [13, 29]. Health facilities in remote areas are not connected to the national grid and have to depend on solar powered equipment or generators. Power outages are common, and some health facilities do not have generators. This limits the use of electricity to run laboratory equipment, and maintain cold chain. Unavailability of running water also limits adherence to infection, prevention and control guidelines in health facilities. The infrastructure improvement is ongoing but need to be fast tracked.

## Conclusion

Good progress has been made in improving health security capacity in Sierra Leone following the 2016 JEE assessment and the NAPHS development processes that followed. A collaborative and facilitative environment in Sierra Leone resulted in significant gains in health security capacity. Operationalization of the annual internal review using the JEE tool and regular online monitoring of NAPHS implementation has enabled the Government of Sierra Leone and partners to modify and support the plan more rapidly. However, much more still needs to be done to ensure that all indicators reach the level of demonstrated and sustainable capacity. The government and development partners should continue to provide leadership and support in NAPHS implementation to ensure that more gains are achieved in the coming years.

## Data Availability

The datasets used/analyzed for this study are available from the corresponding author upon reasonable request.

## Abbreviations

AAR: After Action Review
AMR: Antimicrobial Resistance
AOP: Annual Operational Plan
DHSE: Directorate of Health Security and Emergencies
EPRRG: Epidemic Preparedness, Response, and Resilience Group
EVD: Ebola Virus Disease
GHSA: Global Health Security Agenda
HCAI: Health Care Associated Infection
IHR: International Health Regulations
JEE: Joint External Evaluation
MDRO: Multi-Drug Resistant Organism
MOHS: Ministry of Health and Sanitation
NAPHS: National Action Plan for Health Security
NFP: National Focal Point
REMAP: Resource Mapping
SARS: Severe Acute Respiratory Syndrome
SPAR: State Party Annual Report
US CDC: United States Centers for Disease Control and Prevention
WASH: Water, Sanitation and Hygiene
WHA: World Health Assembly
WHO: World Health Organization
